# Granular Cell Tumour of the Breast: A Rare Mimicker of Carcinoma

**DOI:** 10.7759/cureus.99008

**Published:** 2025-12-11

**Authors:** Shiveta Razdan, Adhrit Jha, Manya Giri Nishad, Arupparna Sengupta

**Affiliations:** 1 Breast Surgery, Amrita Hospital, Faridabad, IND; 2 Pathology, Amrita Hospital, Faridabad, IND

**Keywords:** breast cancer, breast surgery, granular cell tumour, granular cell tumour of the breast, tumours of the breast, wide local excision

## Abstract

Granular cell tumour of the breast is an uncommon benign neoplasm that can closely mimic carcinoma on clinical examination and imaging. We describe a 60-year-old woman who presented with a painless palpable lump in the upper inner quadrant of the left breast. Mammography demonstrated an irregular mass categorised as Breast Imaging-Reporting and Data System (BIRADS) 4, and ultrasonography was BIRADS 5. Breast magnetic resonance imaging (MRI) showed a 27×20 mm irregular, spiculated, heterogeneously enhancing lesion. Ultrasound-guided core biopsy established the diagnosis of granular cell tumour. The patient underwent breast-conserving wide local excision, and final histopathology revealed a poorly circumscribed tumour composed of cells with abundant eosinophilic granular cytoplasm, with negative margins and no atypical features. Postoperative recovery was uneventful. Surveillance mammography at 12 months was unremarkable, and at 24 months, the patient remains asymptomatic with a normal clinical breast examination and no evidence of recurrence. This case underscores the potential for granular cell tumour to imitate malignancy and highlights the importance of imaging-pathology concordance to avoid overtreatment. When a tissue diagnosis is secured preoperatively, breast-conserving surgery with margin negativity is usually sufficient, and routine clinical and age-appropriate imaging follow-up can document durable control. Our patient's two-year disease-free course supports a conservative, margin-oriented approach to management in similar presentations.

## Introduction

Granular cell tumours (GCT) can rarely originate in the breast, constituting 5-6% of all reported cases [1,2], and occur in approximately one in every 1,000 breast cancers [3]. Often seen in the premenopausal age group, GCT of the breast predominantly affects women of African American ancestry [4]. With advancement in immunohistochemistry, the origin of these tumours has been located to perineural cells, which is supported by their S100 protein positivity, similar to Schwann cells [5,6]. Though usually benign, these tumours mimic scirrhous breast carcinoma not only clinically and radiologically but also on frozen section. This can lead to misdiagnosis and further unnecessary radical treatment. Hence, the diagnosis of this tumour can be made by a combination of core biopsy and immunohistochemistry, as we demonstrate in this case report.

## Case presentation

**Figure 1:**
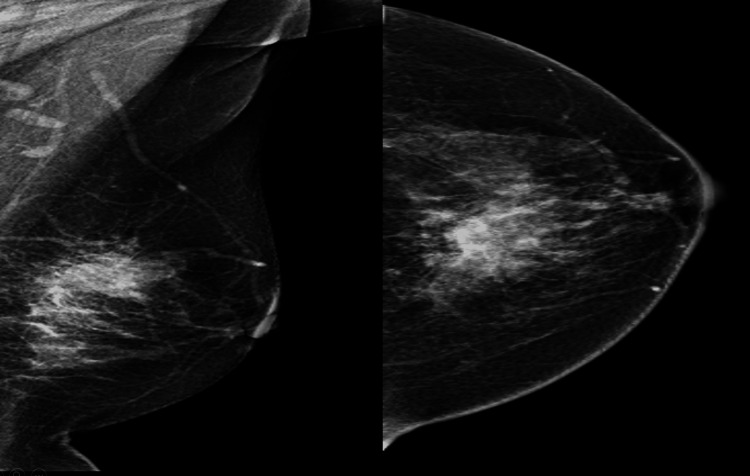
Mammogram (CC and MLO views) showing irregularly shaped high-density mass with spiculated margins in the upper inner quadrant of the left breast CC: craniocaudal; MLO: mediolateral oblique

We present a case report of a 60-year-old post-menopausal, non-insulin-dependent diabetic female patient, who had presented to us with a one-month history of a lump in her left breast with no associated symptoms. She denied having a family history of breast cancer and had carried two full-term pregnancies. On clinical examination, she was found to have an irregular solitary lump measuring approximately 25 mm in the upper inner quadrant of the left breast. Examination of the right breast was unremarkable. No axillary lymphadenopathy was noted on either side. Mammography was ordered, which showed a Breast Imaging-Reporting and Data System (BIRADS) 4-rated lesion in the upper inner quadrant of the left breast (Figure 1).

**Figure 2:**
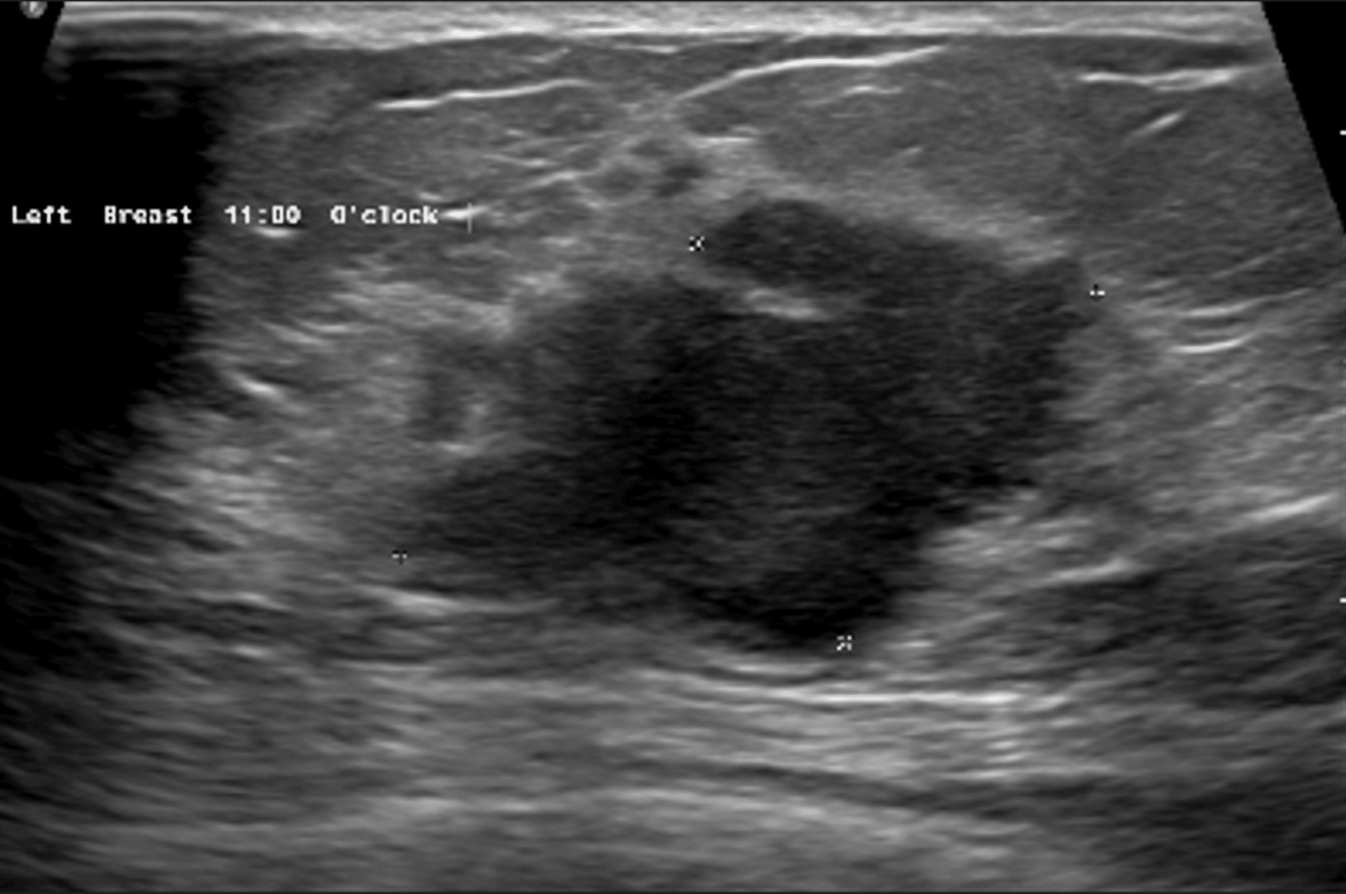
Ultrasonography of the left breast showing a BIRADS 5 lesion in the upper inner quadrant of the left breast BIRADS: Breast Imaging-Reporting and Data System

On ultrasonography, a BIRADS 5-rated 25×18 mm lesion was observed in the upper inner quadrant of the left breast (Figure 2).

**Figure 3:**
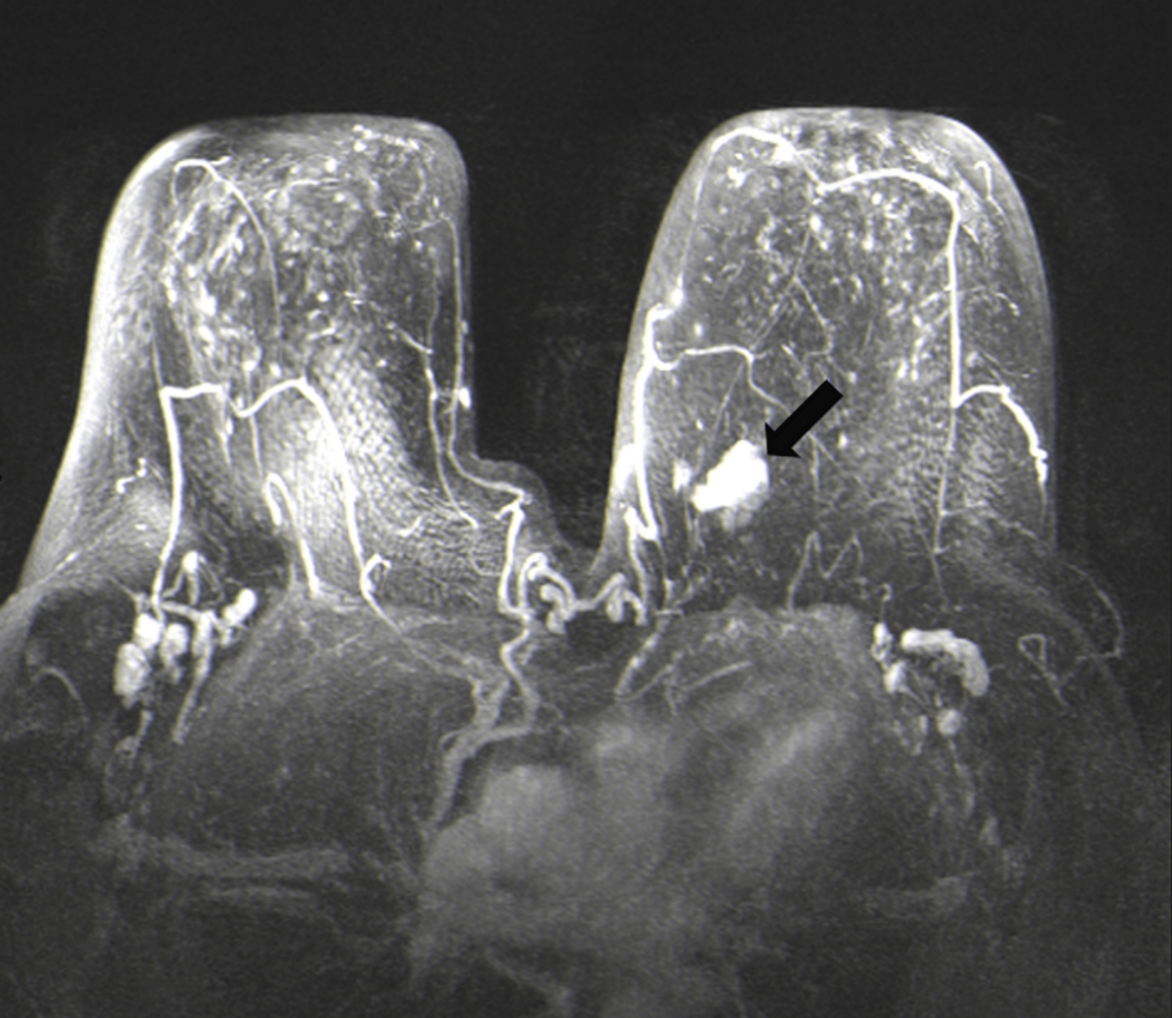
MRI of the breast showing a 27×20 mm solitary irregular lesion (arrow) in the upper inner quadrant of the left breast MRI: magnetic resonance imaging

Contrast magnetic resonance imaging (MRI) of the breast showed a 27×20 mm solitary irregular lesion with tiny spicules in the upper inner quadrant of the left breast with heterogeneous enhancement (Figure 3).

**Figure 4:**
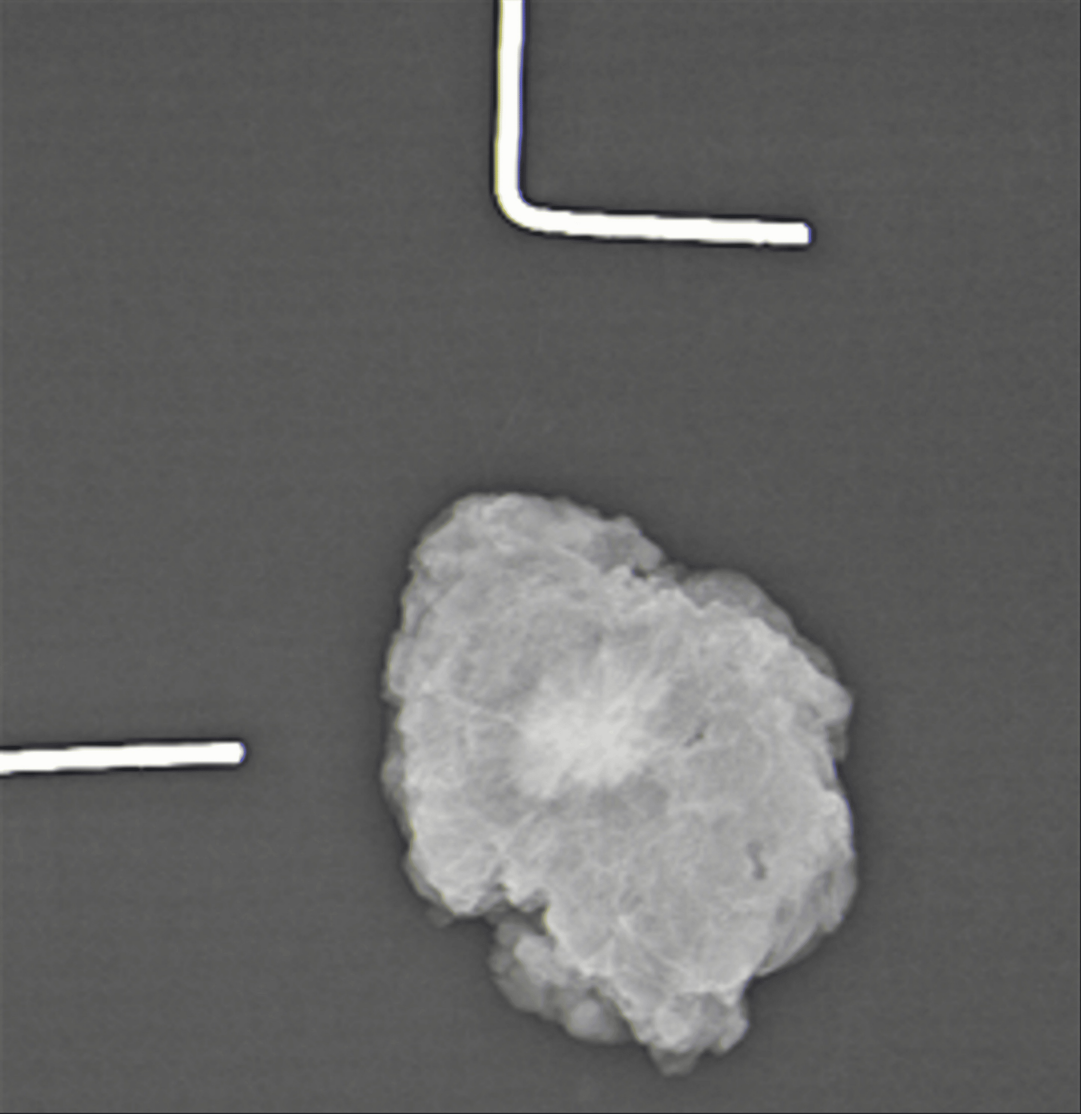
Mammogram of the specimen showing the lesion with clear margins

Keeping the radiological picture in mind, the patient underwent a USG-guided core biopsy, which confirmed the lesion to be a GCT. She then underwent wide local excision of the left breast lump. Specimen mammography showed the complete removal of the tumour with margins (Figure 4).

**Figure 5:**
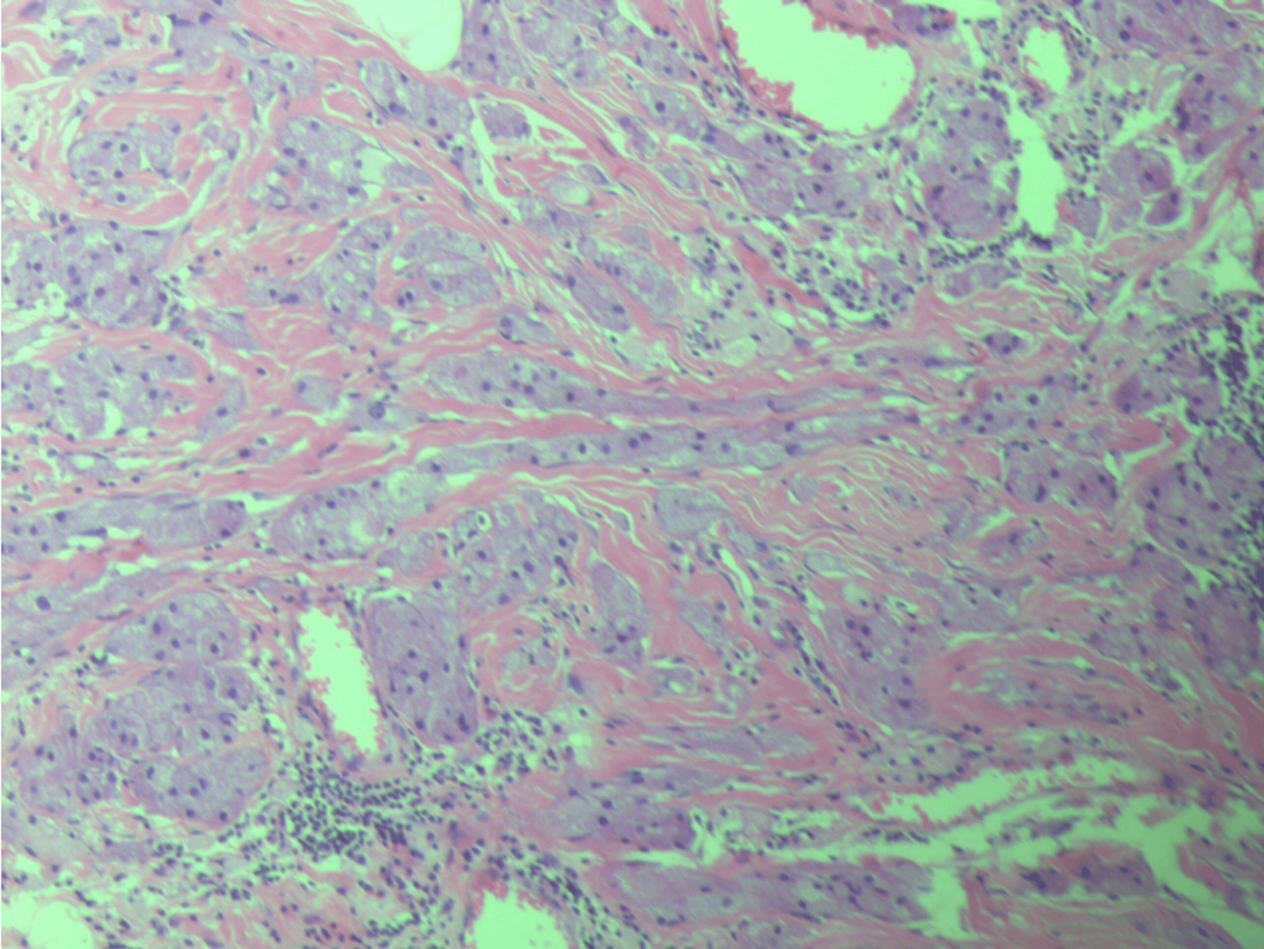
Individual cells with abundant eosinophilic granular cytoplasm

She was discharged on the next day of surgery. Final histopathology showed a poorly circumscribed lesion composed of cells infiltrating the breast tissue. Individual cells showed abundant eosinophilic granular cytoplasm, and nuclei were small with no evidence of malignancy (Figure 5).

**Figure 6:**
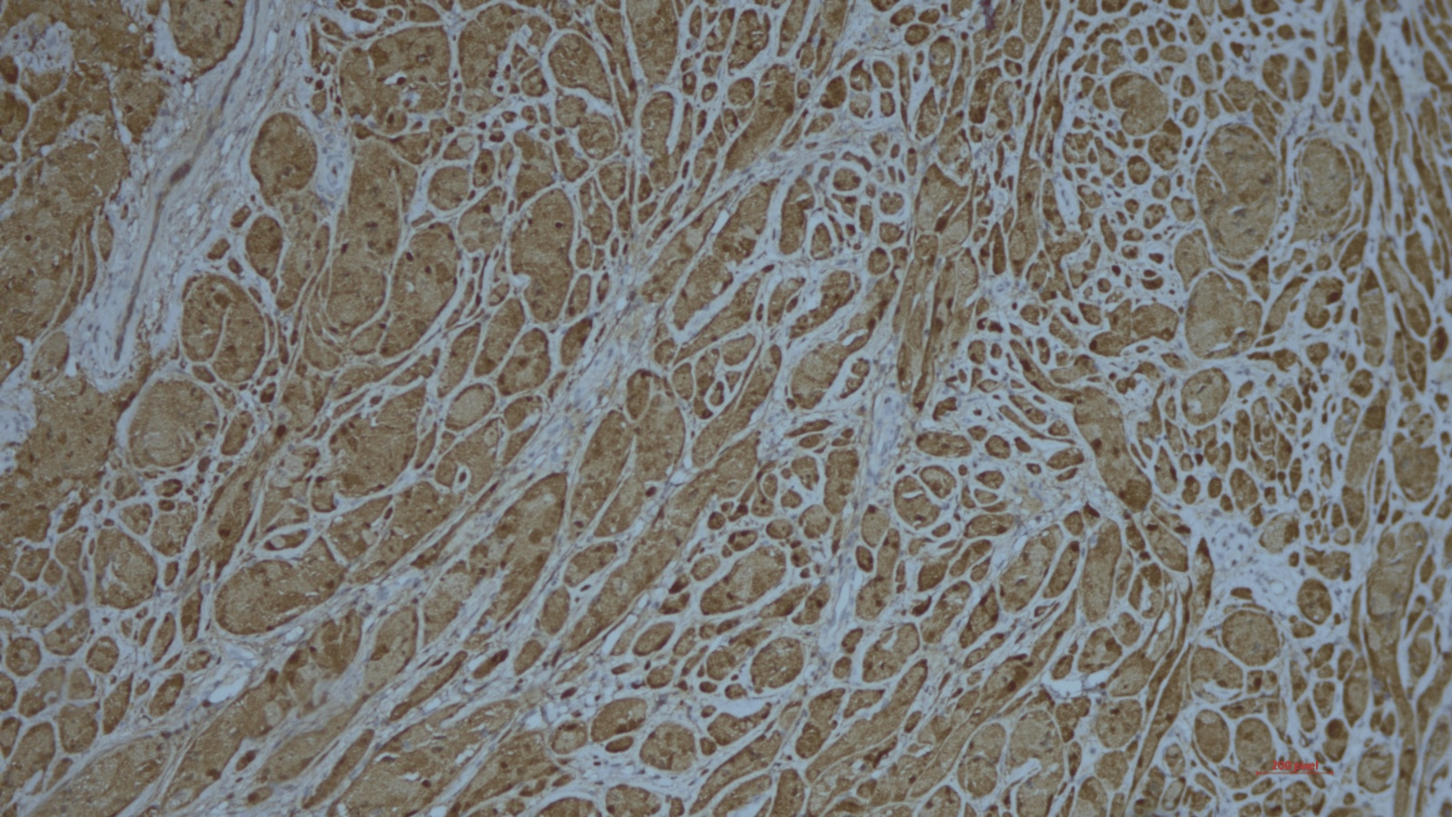
S100 positivity seen on immunohistochemical staining

On immunohistochemical staining of the sections, diffuse positivity for S100 was seen in tumour cells (Figure 6).

Resection margins were also reported to be free. Surveillance mammography at 12 months showed no residual or recurrent lesion, and at 24 months, the patient remained asymptomatic with a normal clinical breast examination and no clinical evidence of recurrence.

## Discussion

The first GCT was reported by Abrikossoff in 1926, who described it as a myoblastoma [7]. It commonly occurs in the subcutaneous tissue of the head and neck region and frequently involves the tongue, though it can occur at various other body sites as well [8]. GCT of the breast can often mimic breast carcinoma, due to its hard consistency, along with the fact that it may be adherent to the underlying muscle with skin retraction. Patients usually present with a painless, mobile mass in the upper inner quadrant. This characteristic location in the upper inner quadrant has been attributed to innervations of that area by the supraclavicular nerve, as was also seen in our case [2]. The presentation of these lesions on mammography may range from a well-circumscribed mass to an indistinct lesion, leading to suspicion for malignancy, and on ultrasonography, an ill-defined solid mass with posterior acoustic shadowing or enhancement can often be seen [9].

Grossly, the tumour presents predominantly as yellow-white with a scirrhous pattern often mimicking carcinoma, whereas histologically, the characteristic presentation is of cells with abundant granular and eosinophilic cytoplasm from which this tumour derives its name and nuclei that are small, round, uniform, and deeply basophilic; mitoses and pleomorphism are generally absent [10].

Malignancy in GCT is exceptionally rare and accounts for about 1-2% of reported cases [11]. A large study conducted in 1998 by Fanburg-Smith et al. showed that malignant GCT should be favoured when ≥3 of the following histological features are present: (1) necrosis; (2) spindling of tumour cells; (3) vesicular nuclei with prominent nucleoli; (4) increased mitotic activity (>2 mitoses/10 high-power fields at 200× magnification); (5) a high nuclear-to-cytoplasmic ratio; and (6) marked nuclear pleomorphism [12].

Differential diagnosis can be challenging, with lesions like breast carcinoma, sclerosing adenosis, and histiocytic or metastatic lesions all being candidates. Management involves a wide excision of the tumour. There is no role for chemotherapy or radiotherapy, though local recurrence can occur if not excised completely.

## Conclusions

Though GCT of the breast is a benign condition, it should be investigated properly to differentiate it from malignancy to avoid radical treatment. Definite diagnosis is by immunohistochemistry. Complete excision with negative margins achieved durable control in our case, with the patient being disease-free at 24 months.
